# A Study of Variations of the Stomach in Adults and Growth of the Fetal Stomach

**DOI:** 10.7759/cureus.28517

**Published:** 2022-08-28

**Authors:** Azra M Karnul, Chaitanya K Murthy

**Affiliations:** 1 Anatomy, East Point College of Medical Sciences and Research Centre, Bengaluru, IND; 2 Anatomy, Hassan Institute of Medical Sciences, Hassan, IND

**Keywords:** diameter of pyloric sphincter, rugae, growth of fetal stomach, iugr, pyloric sphincter, pylorus, lesser curvature, greater curvature, stomach

## Abstract

The stomach is a site for various pathological conditions like congestive hypertrophic pyloric stenosis, peptic ulcer, gastroesophageal reflux disease (GERD), and carcinoma of the stomach. Further, for the treatment of obesity too, surgical manipulation of the stomach is done by a bariatric surgeon. With the availability of a wide range of diagnostic tools like barium meals, USG, CT scan, MRI, and endoscopy, it is possible to identify the variations in the position and shape of the stomach and developmental defects while diagnosing diseases. As thorough knowledge of stomach position and variations will help in preoperative planning and preventing inadvertent damage during surgeries, this topic was taken up for research.

Aims and objectives

This study aims to study the variations of the stomach in human cadavers and dead fetuses with regard to its length, shape, capacity, ends, curvatures, and mucosal folding and classify them into various groups. In addition, this study also aims to assess the pattern of growth of the stomach in fetuses.

Material and methods

The stomachs of 50 adult cadavers and 20 dead fetuses were studied by standard dissection method, concerning their topography, shape, level of the cardiac and pyloric orifice, cardiac angle, length of greater (GC) and lesser curvatures (LC), pyloric sphincter, volume, and mucosal folds.

Results

The stomach was located in the left hypochondriac quadrant in 78% of the samples and in relation to the 7th costal cartilage in 64%. The two main types of classification established were Type I (variation in position along the vertical axis) in 4% and Type II (variation in position along the transverse axis) in 14%. Type III classification comprised the variations in shape, with a J-shaped stomach in 58%, cylindrical in 20%, crescentic in 14%, and reversed L in 8%. The average length showed significant differences in males, 19±2.48 cm vis-a-vis females, 17.1±2.01 cm. In 66% of the cases, the cardiac orifice was to the left of the midline behind the 7th costal cartilage, and the pyloric orifice was to the right, 1.2 cm to the midline and in the transpyloric plane in 76%. The average GC and LC were 33.6±1.43 cm and 27±5.28 cm, respectively. GC was more significant in males. The average length and diameter of the pyloric canal were about 3.56±0.38 cm & 0.77±0.23 cm, respectively. The thickness of the pyloric sphincter did not show a significant gender difference. The average volume was 289.88±69.15 ml. Rugae were normally spaced in 68%, nearly spaced in 18%, and widely spaced in 6%. The fetal stomach measurements were significantly correlated to gestational age and showed linear growth.

Conclusion

The study of the morphology of the stomach and its variations are important not only to surgeons and anatomists but also to gastroenterologists. The linear growth of the stomach in embryos helps radiologists and obstetricians to diagnose intrauterine growth retardation (IUGR) and congenital anomalies early.

## Introduction

The stomach is situated in the upper abdomen, the left upper quadrant, which is the left hypochondriac, and extends downward, forward, and to the right epigastric and umbilical areas. It occupies a recess beneath the diaphragm and anterior abdominal wall, which is bounded by the upper abdominal viscera on either side and in front of the transverse colon. The stomach develops in the distal part of the foregut as a slight fusiform dilatation initially in the median plane. This primordia of the stomach enlarge and broadens ventrodorsally, more so at the left wall of it. During the next two weeks, on account of the re-arrangement of the epithelium, the dorsal border grows faster than its ventral border, which demarcates the development of greater curvature of the stomach (GC). Thereafter passive rotation of the stomach and intestine leads to a change in position and relocation of the stomach. Various factors influence the shape and position of the stomach, including the posture and build of the individual, the position of the surrounding viscera, the tone of the abdominal wall, gastric musculature, and mainly, the extent of filling of the stomach because the pyloric part usually remains contracted, and the stomach expands downward and forward. When intestinal distension interferes with the downward expansion of the body of the stomach, the stomach retains a horizontal position [1]. The empty stomach is most commonly J-shaped, the fundus usually contains gas and is erected, and the pylorus descends to the level of the second or the third lumbar vertebra. The lowest part of the antrum often lies below the level of the umbilicus, and the axis of the organ is obliquely placed [1]. In short, in obese individuals, the axis of the stomach lies more towards the horizontal as a "steer-horn" shape. Such people are prone to suffer from duodenal ulcers, and in tall and thin individuals, the axis of the stomach is vertical, and they are susceptible to gastric ulcers [2]. In new-born and children, due to the relatively larger liver, the stomach will be more vertical and on the left side. Morphologically, the variations of the stomach are classified into five types in the CT scan study: I: variation in position along the vertical axis; II: variation in position along the transverse axis, which is characterized by gastric cascade (hourglass stomach); III: abnormal shapes; IV: congenital connections like fistulas (nonpathological); and V: mixed variety (which involve two or more groups from I-IV) such as malrotations and diaphragmatic hernia [3].

## Materials and methods

This study was conducted with 70 samples, comprising 50 adults and 20 fetuses. There were 48 males and eight females in adults and six males and 14 females in fetuses. The adult cadavers were embalmed cadavers placed for undergraduate dissection in the department of anatomy and post-mortem bodies in the forensic departments of Navodaya Medical College and Research Center, Raichur. The dead fetuses were collected from the OBG department of the same medical college hospital and were freshly fixed with 10% formalin through the umbilical artery. Dissection was done as per the guidelines of the Cunningham Manual of Dissection. Two incisions were made, one passing from the xiphoid process through the umbilicus to the pubic symphysis and the other extending from the pubic symphysis to the anterior superior iliac spine on either side. The flaps of the skin were reflected, leaving the superficial fascia on the anterior abdominal wall. The superficial fascia was divided in the midline. The external oblique, transverse abdominis muscles, and rectus abdominis muscles were identified and incised in the vertical and the horizontal planes. The structures in the peritoneal cavity were visualized. The greater momentum was identified, and its continuity with the stomach was noted. The topography of the stomach was noted as in situ. The levels of the cardiac and pyloric orifices were noted. The pyloric sphincter was noted below the pyloric antrum. The thickness was measured with vernier calipers, and the stomach was removed by ligating and incising the gastroesophageal junction and just distal to the pyloric sphincter. After removing and washing the stomach, the cardiac end was ligated. Through the pyloric end, water was injected. Water was taken into the measuring jar, and the volume was noted. The length of the stomach was taken from the highest point of the fundus to the point on the line joining the incisure angularis and GC. The width was measured from the highest point of the body to the lesser curvature (LC). The length of the GC was taken from the cardiac orifice along the left border of the pyloric orifice. The length of the LC was taken from the cardiac orifice along the right border to the pyloric orifice. The stomach was opened through an incision along the GC, and the mucosa was studied through a hand lens. The stomach serosa was removed by the window dissection method to study the musculature.

In post-mortem cases

An incision was made from the xiphisternum to the symphysis pubis, the abdominal cavity was opened, and then a procedure identical to the aforesaid was carried out in the mortuary room.

In fetal cases

The preserved fetuses were kept in a supine position. A midline incision was done from the xiphisternum to the symphysis pubis and a horizontal incision at the level of the umbilicus. The anterior abdominal wall was reflected in the form of four flaps. In fetuses, it was observed that the anterior surface of the stomach was covered by the left lobe of the liver, which extended nearly as far as the spleen, and only a small portion of the GC of the stomach was visible anteriorly. The left lobe of the liver was incised, and details of the stomach were observed, as for the adult specimens. All measurements were calculated by thread and vernier calipers, and the cardiac angle was measured through a goniometer by drawing a horizontal line from the short gastric artery to a point on the cardiac orifice where the esophagus meets the GC of the stomach. The volume was measured in the same way as for adult cadavers. The anthropometric measurements and variations of the stomach were expressed as proportions or percentages and mean ± SD. Statistical analysis was performed with IBM SPSS statistics version 16.0. The Pearson correlation coefficient was calculated to examine the pattern and correlation between the gestational age and GC, LC, and volume of the stomach.

## Results

**Figure 1:**
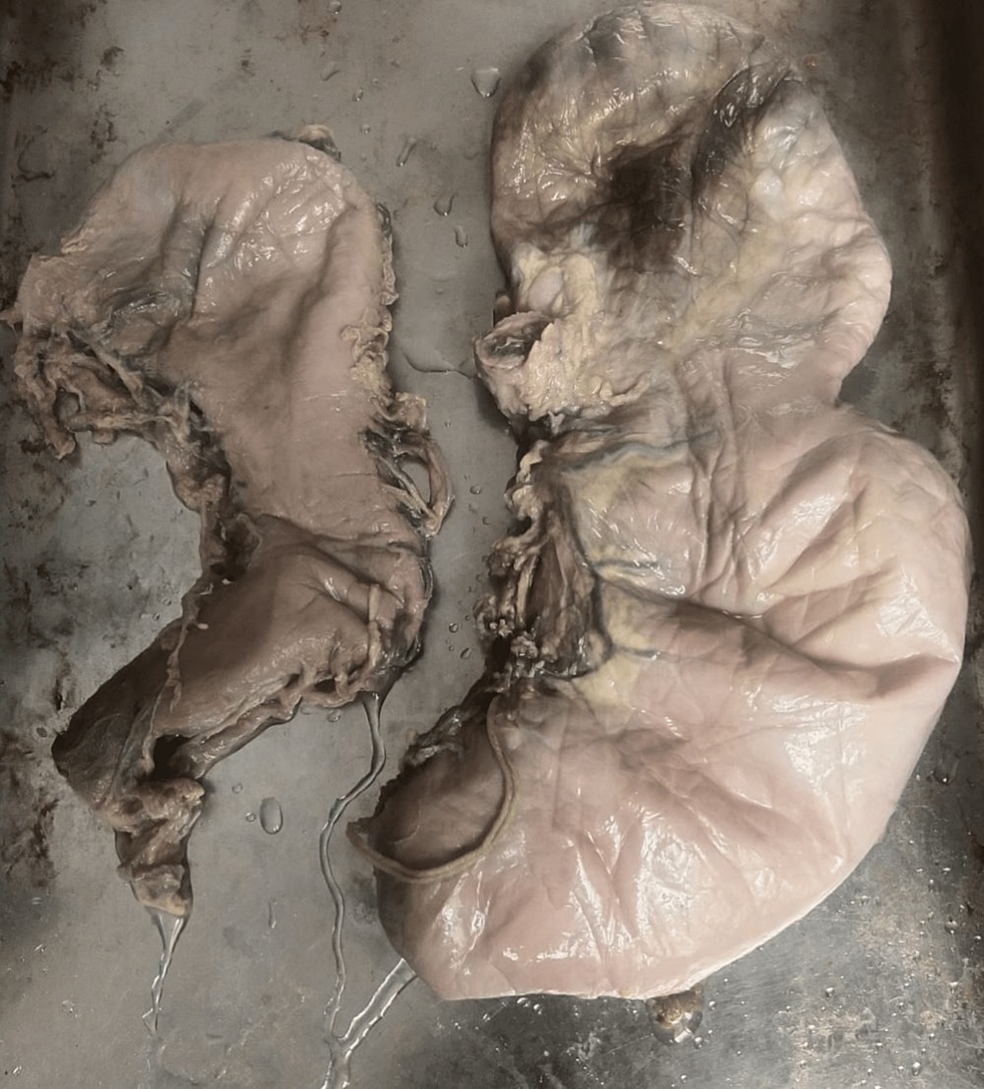
Type II: abnormal positioning along transverse axis (hourglass stomach).

**Figure 2:**
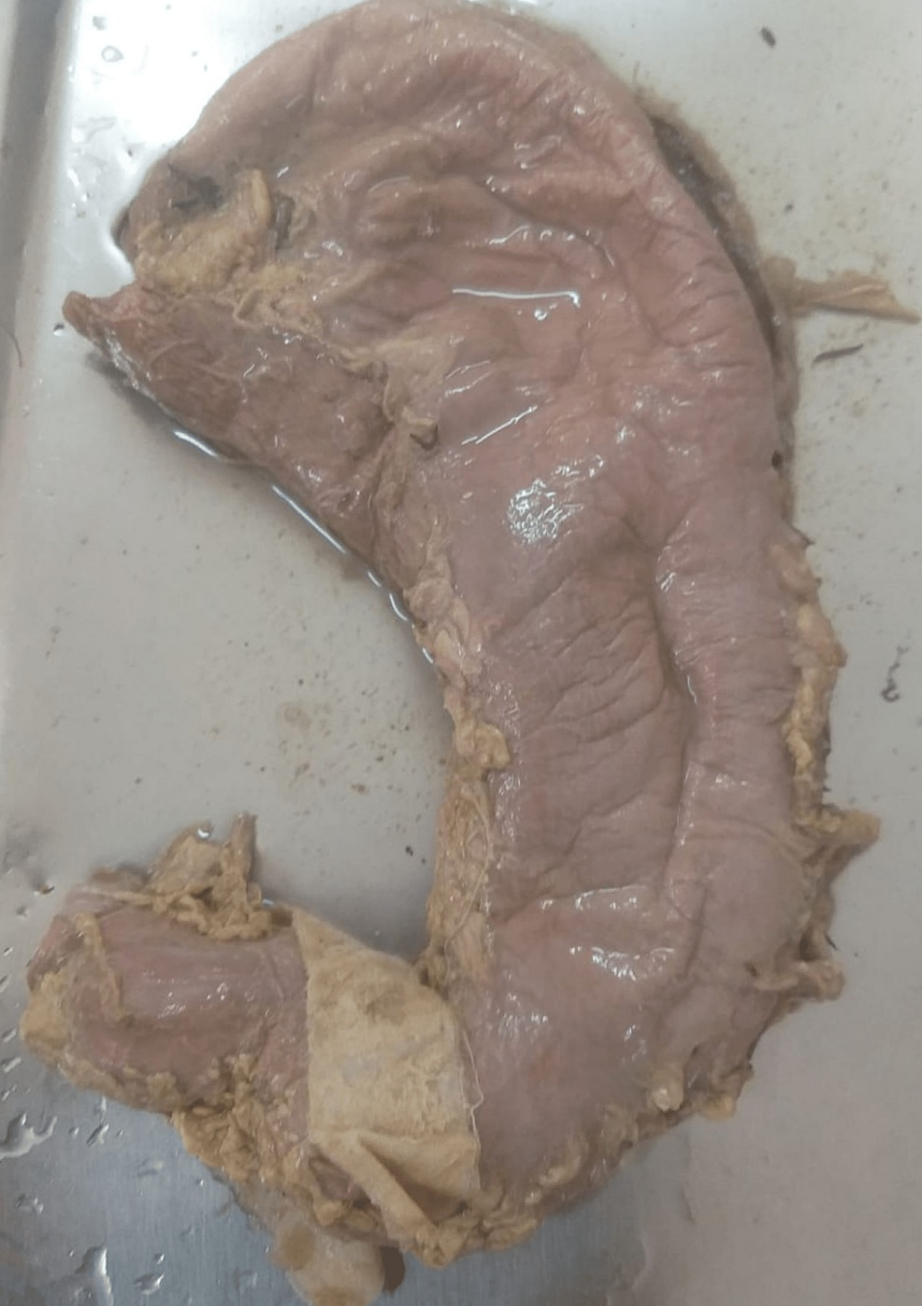
Type III: variation according to shape (J shape stomach).

**Figure 3:**
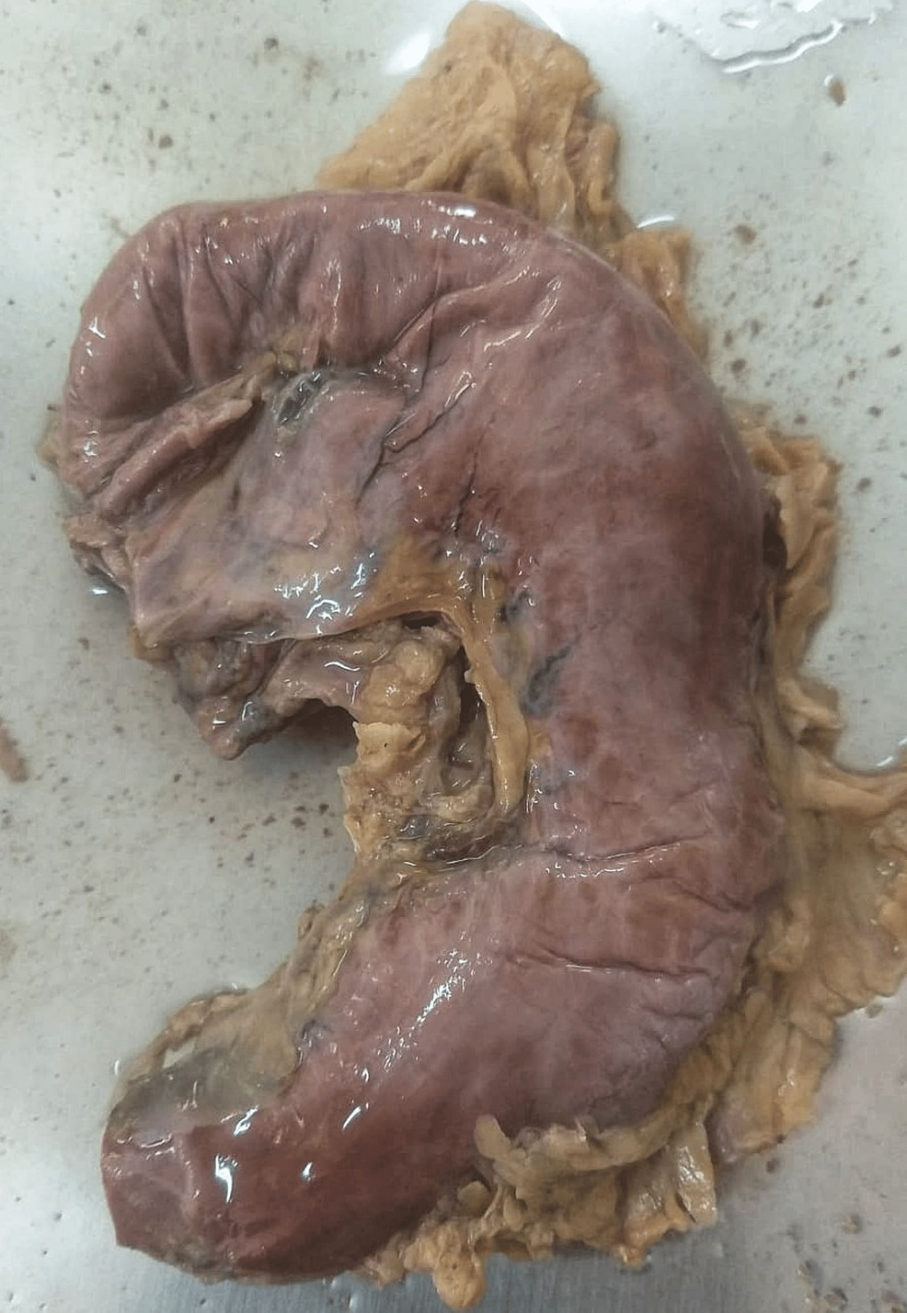
Type III: variation according to the shape (crescentic stomach).

**Figure 4:**
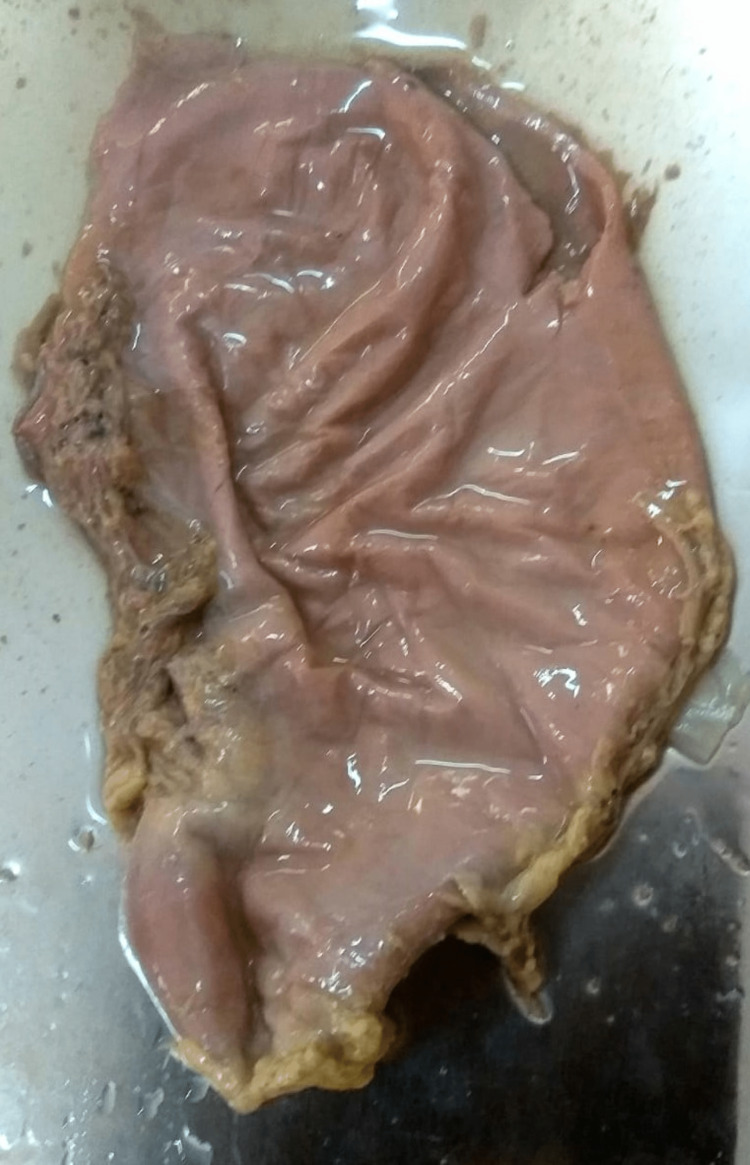
Type III: variation according to the shape (dilated stomach).

**Figure 5:**
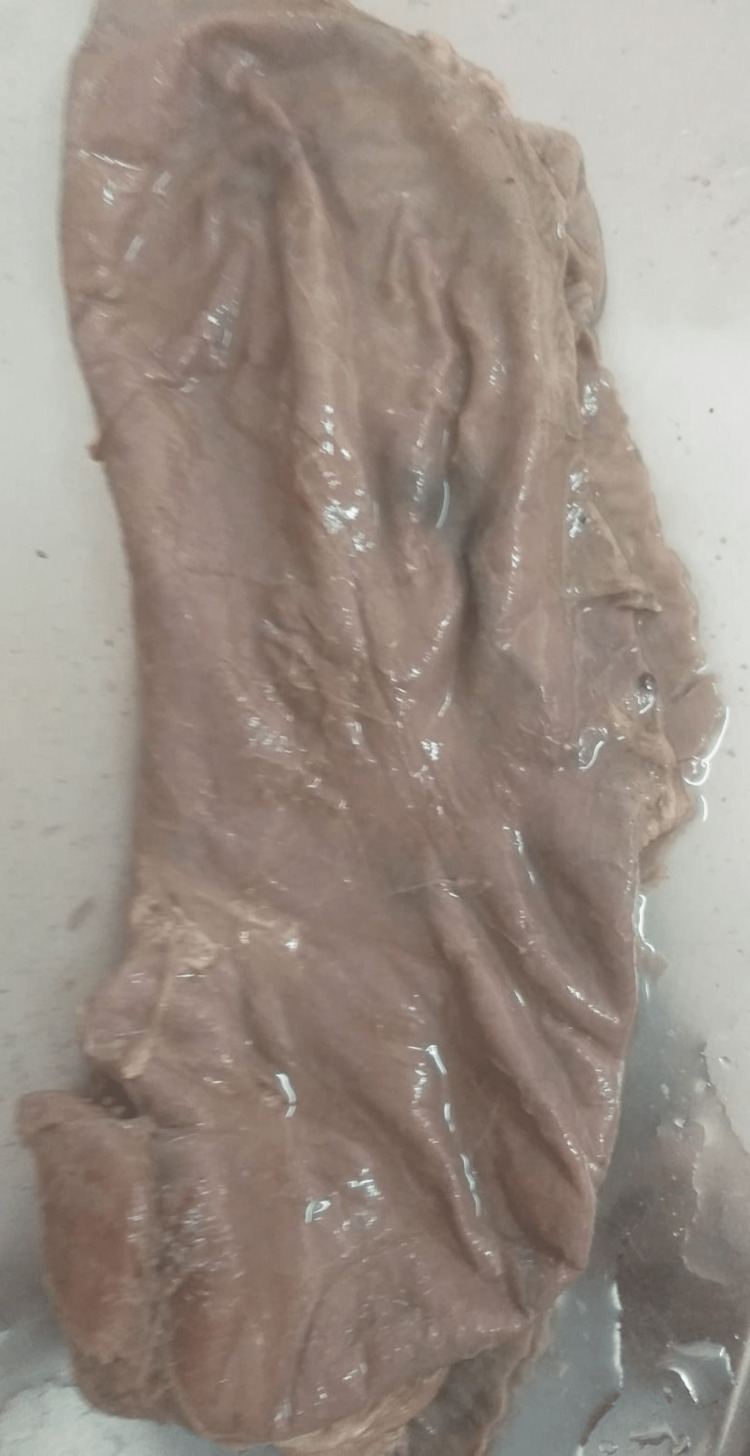
Type III: variation according to the shape (cylindrical stomach).

**Figure 6:**
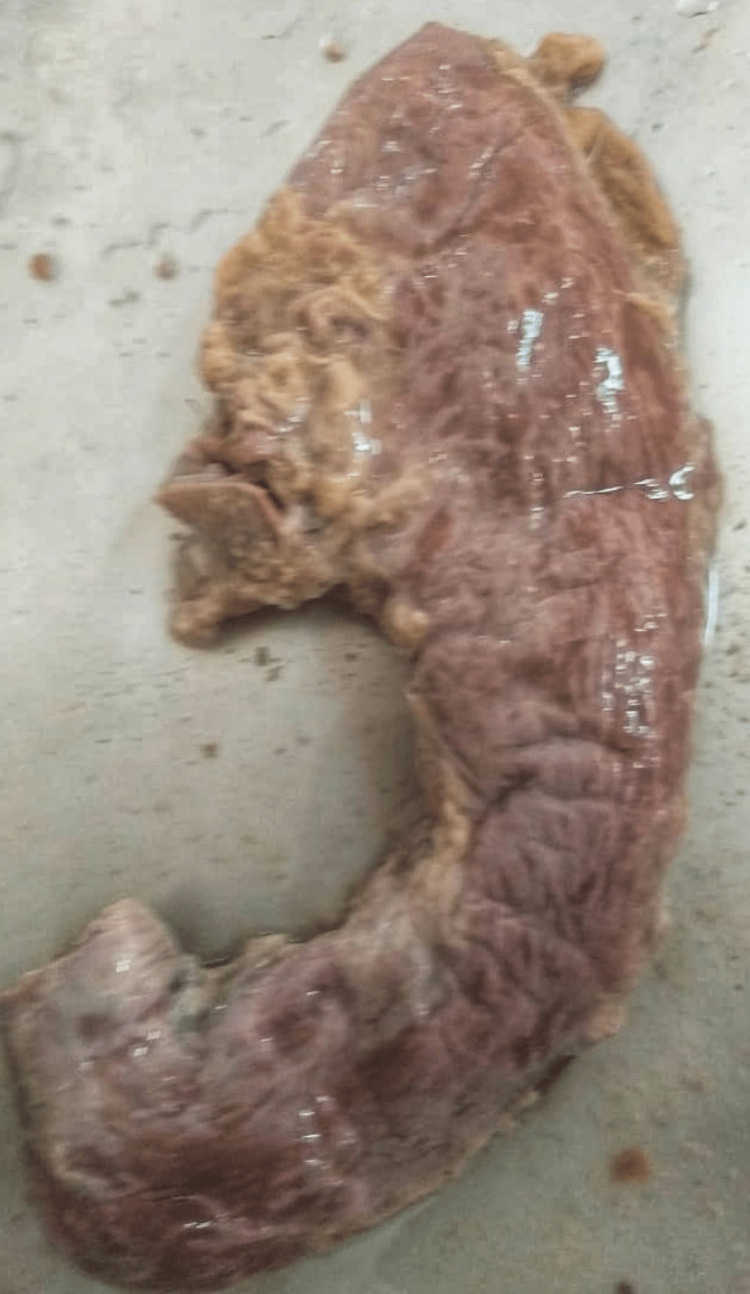
Type III: variation according to the shape (reversed L shaped stomach).

The topography of the stomach most commonly observed was in the left hypochondriac area (78%), the epigastric region (74%), the seventh costal cartilage (64%), 62% in relation to the left lobe of the liver and spleen; it was 38% above the umbilicus, 34% in relation to the umbilicus, and 18% below the umbilicus. As shown in Table 1, according to the classification of Burdan F et al., the following variations were observed: Type I, where the variation in position is along the vertical axis, and Type II, where the variation in position is along the transverse axis; the stomach was divided into two pouches with a constriction in between called as hourglass stomach (Figure 1). The interior of such stomachs did not show any discoloration or ulcers. As for Type III (variations in shape), the stomach was J-shaped in 58% of the specimens as shown in Figure 2, cylindrical in 20% as shown in Figure 3, Crescentic in 14% as shown in Figure 4, Dilated in 10% as shown in Figure 5, and Reversed L in 8% as shown in Figure 6. Type IV and Type V were not noted. The shape of the GC was normal/curvilinear in 85% and straight or hockey stick-shaped in 18%. The LC was more concave in 58% and straight/curvilinear in 42% of the cases. The average intragastric volume of the stomach was about 289.88±69.15 ml, the majority being in the range of 250-300 ml. The difference in the capacity of the stomach between both gender was not significant.

**Table 1:** Classification of variations of the stomach. LC: Lesser curvature.

	Morphology of stomach	Frequency	Percentage (%)
I Abnormal positioning along long axis:	a. LC faces forward or backward or to right	2	4
	b. Translocated to chest cavity	0	0
II Abnormal positioning along horizontal axis:	Hour glass	7	14
III. According to shape	J shaped	29	58
	Cylindrical	10	20
	Short	0	0
	Crescentic	7	14
	Dilated	5	10
	Reverse L	4	8

In males, the length of the stomach was about 14-17 cm in 15%, 18-21 cm in 64%, and 22-25 cm in 21%. Whereas in females, it was 14-17 cm in 38%, 18-21 cm in 50%, and 22-25 cm in 13%. As shown in Table 2, the average length of the stomach was about 19±2.48 cm in males and 17.1±2.01 cm in females, significantly longer in males, with a p-value of 0.01. Table 2 further shows that the average length of the GC was 33.6±1.43 cm, and that of the LC was 27±5.28 cm. The difference between GC and LC was about 6 cm. There was a significant increase in the length of the GC in males compared to females (34±3.48 cm vs. 29.2±2.01 cm with a p-value: 0.003). The length of LC showed no significant variation gender-wise. The position of the cardiac orifice was 66% to the left of the midline at the seventh costal cartilages and in 34% of the specimens, it was to the right of the midline at the seventh costal cartilage. The average cardiac angle was about 63.5±3.54°. A total of 75% of the stomach had a cardiac angle between 60° and 65° and 14% between 70° and 75°.

The position of the pyloric orifice was 1.2 cm to the right of the midline on a transpyloric plane in 76%. Table 2 shows that the length of the pyloric canal in males was about 4.55±0.39 cm and in females, 3.56±0.38. The thickness of the pyloric sphincter was about 5.52±6.18 mm in males and 4.5±0.15 in females, showing no significant difference. In Table 2, the average diameter of the pyloric sphincter was about 7.7±0.23 mm in males and 6.8±0.40 mm in females. Normal mucosa was found in 90% of the specimens, while 10% had pathological mucosa showing the presence of ulcers or perforations. The distribution of rugae, straight at the LC, was present in 46%, curvilinear, and absent in 16%. The rugae were not found in 18% of specimens, normally spaced (0.6-1 mm) rugae were found in 68%, widely spaced (more than 1 mm) in 6%, and less spaced rugae (less than 0.6 mm) were found in 18%. Gastric pits were found in 100% of the specimens.

**Table 2:** Various dimensions of the stomach. Values are presented as frequencies and percentages, mean ± SD, cm: centimeters, P-value: <0.05 is significant. *significant, ** highly significant.

Variables	Males	Females	P-value
Length	19±2.48 cms	17.1±2.01 cms	0.01*
Volume of stomach <150ml	16 (38%)	1 (13%)	
150-250ml	1 (2%)	1 (12.5%)	
250-300ml	23 (55%)	5 (62.5%)	
>300 ml	3 (7%)	2 (25%)	
Greater curvature	34.4±3.48 cms	29.2±2.01 cms	0.003**
Lesser curvature	28.02±2.5 cms	26.05 ±2.3 cms	0.021
Pyloric canal length	4.55±0.39 cms	3.56±.38 cms	0.365
Pyloric sphincter thickness	5.2±6.18 mm	4.5±0.15 mm	0.51
Diameter	7.7±0.23 mms	6.8±0.40 mms	0.416
Rugae	Present: 4 (82%)	Absent: 9 (18%)
Normally spaced	34 (68%)
Closely placed	3 (6%)
Distantly placed	9 (18%)

In fetuses

Fetuses taken were aged 16-40 weeks of gestation (mean gestational age 29). The mean lengths of GC and LC in fetuses were 63.24±22.34 and 22.37±7.60 mm, respectively, with a difference of about 3 mm between GC and LC. The mean capacity of the stomach was 17.84±7.06 ml. GC, LC, and volume were significantly correlated with the gestational age of fetuses, as shown in Table 3.

**Table 3:** Correlation between gestational age, greater curvature, lesser curvature, and volume in fetuses. ** Correlation is significant at 0.01 level (2-tailed).

		Gestational Age (in Wks)	Greater Curvature (in mm)	Lesser Curvature (in mm)	Volume (in ml)
Gestational Age (in Wks)	Pearson Correlation	1	0.991^**^	0.975^**^	0.971^**^
	Sig. (2-tailed)		0.000	0.000	0.000
	N	19	19	19	19
Greater Curvature (in mm)	Pearson Correlation	0.991^**^	1	0.985^**^	0.973^**^
	Sig. (2-tailed)	0.000		0.000	0.000
	N	19	19	19	19
Lesser Curvature (in mm)	Pearson Correlation	0.975^**^	0.985^**^	1	0.972^**^
	Sig. (2-tailed)	0.000	0.000		0.000
	N	19	19	19	19
Volume (in ml)	Pearson Correlation	0.971^**^	0.973^**^	0.972^**^	1
	Sig. (2-tailed)	0.000	0.000	0.000	
	N	19	19	19	19

**Figure 7:**
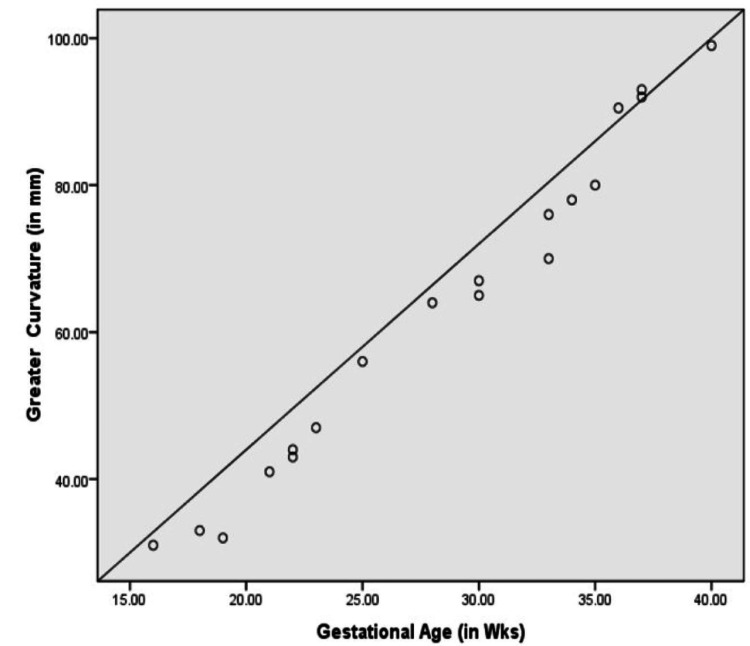
Correlation between gestational age and greater curvature.

**Figure 8:**
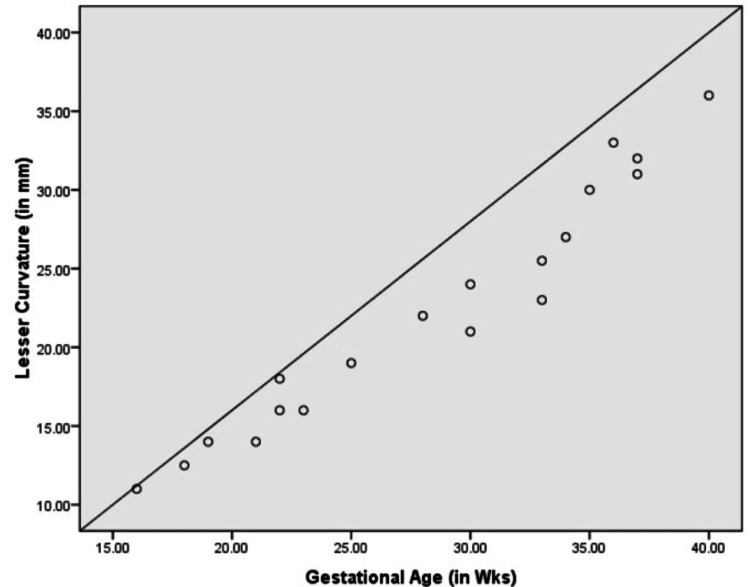
Correlation between gestational age and lesser curvature.

**Figure 9:**
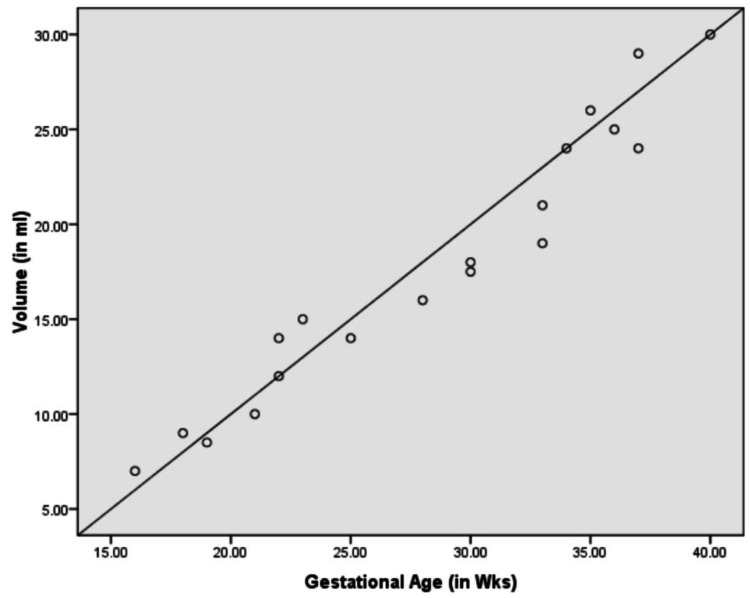
Correlation between gestational age and volume.

Figures 7-9 show a graphic presentation of the relation between gestational age of fetuses with GC, LC, and volume of fetal stomachs showing the linear growth. None of the fetuses showed any variations in position along the vertical or horizontal axes, and variation in shape was also not observed.

## Discussion

The variation of the location of the stomach depends on food habits, growth, build and posture. In an erect posture, it is either vertical or slightly inclined towards the right. In the supine position, it is more oblique, placed with the axis directed forward [4]. Incisura angularis is more evident in the supine posture. Despite the radiological and surgical importance of the stomach, classification of its anatomical variations has been rarely reported. This study was conducted to review and compare the position with the existing classification.

Hiatal hernia is a geographical and socioeconomic factor-dependent variation reported as the most common variation along the long axis, included in type I variation, including congenital stomach malrotation [3]. We did not encounter such variation in our data. A change in the position of GC, LC, and the pylorus describes the rotation of the stomach. An abnormal position around the transverse axis is described as type II variation. We observed that 14% of the stomach was showing outpouching of the wall of the stomach with a constriction where there is continuity between both cavities. It is reported as biloculation of the cavity of the stomach into a ventral and dorsal recess which could be congenital, acquired, or due to peritoneal adhesions of the stomach and surrounding organ [3]. A horizontally placed stomach is frequently found on X-ray examinations of infants, and after infancy, it acquires a vertical position [3]. Among the various shapes of the stomach (type III), we found J-shaped to be the most common at 58%, similar to the study by Yesupadam K et al. [5], followed by cylindrical at 20%, crescentic at 14%, and reverse L at 8%. Netter FH, Chakraborty NC, and Chakraborty D observed straight or hockey stick-shaped stomachs in asthenic-built and malnourished individuals [6,7]. The crescentic shape is present in individuals with hypertrophy associated with pyloric stenosis with or without a dilated stomach [7]. In the present study, in 18% of the specimens, the greater curvature was straight/hockey stick-shaped. In 85%, it was curvilinear in shape. Aforesaid variation in position and shape could be congenital or due to diseases of the stomach or surrounding organs in which the stomach acquires functional changes with/without symptoms [3]. Related to these variations, specific symptoms are not much known; therefore, the incidence of these variations is not well investigated. Burdan F et al. reported (type IV and V) connections/fistulas and mixed forms of variations [3]. We did not find any such variation in our study, which could be due to the involvement of cases admitted for esophageal and stomach surgeries in their study.

The stomach's capacity varies from 30 ml at birth, increases to 1000 ml at puberty, and 1500 ml in adults [1]. In the present study, the capacity/volume of the stomach was reported to be reduced because of the fixation of the stomach in formalin, which generally decreases the volume and length of the organ. However, a radiological study by Lamart S et al. also showed a similar volume at 372 ml [8]. BMI, food habits, and the filling of the intestines are the factors that influence the volume of the stomach. Smaller stomach capacity is present in cases of malnutrition or poor food habits. Our study also showed 10% dilated stomachs. The average length of the stomach is 22-23 cm, and the average width is about 14-15 cm, similar to the study of Lamart S et al. [8]. The maximum length of the GC was about 42-43 cm. The least length was about 28-29 cm. The maximum length of LC was 34-37 cm, and the least length was about 10-13 cm. These findings could change the angle of the cardiac orifice. The maximum length and width may be due to obesity, regional difference, and short stature. The above findings also signify the nutritional status of an individual, food habits, stature, and frequency of food intake. The stomach location and size are dependent on the body weight of individuals [8]. The GC is directed mainly forward and is 4-5 cm longer than the LC [9]. Torgerssen J observed that the filling of the stomach increased the length of the gastric curvatures and the proximal part of the stomach [10]. GC showed gender specificity with a p-value of 0.003, indicating that GC is significantly increased in males compared to females. Similar findings were reported by Lee EG et al., and it was concluded that the length and GC of the stomach were more in males and affected by the BMI of patients [11]. In contrast, Csendes A and Burgos AM reported that the length of the GC is reduced in high morbid cases compared to controls. A gender difference was not noted in their study [12].

In our study, the cardiac orifice was to the 2.5 cm left of the midline behind the seven costal cartilage, similar to the findings of other researchers [7,13]. However, in our study, in 34% of the cases, it was to the right of the midline. This variation could be due to intrinsic factors and space-occupying lesions like malignancy or lymphadenopathy, causing a push or pull to one side. In the present study, the average cardiac angle was about 63.5°, and in 14%, it was 70-75º. An acute angle produces a valve-like effect at this orifice where no anatomical sphincter prevents the reflex. The variation in the angle may be due to the enlarged stomach. A greater angle denotes a greater length of GC and associated stomach enlargement. Wald Hánd Polk HC Jr reported that the cardiac angle varied according to the stomach size [14].

In the present study, in 76% of the specimens, the pyloric orifice was 1.2 cm to the right of the midline of the transpyloric plane, correlating with the other studies [7,13]. In 24% of the specimens, the pyloric orifice was towards the left of the midline, which could be due to the deviation of the stomach due to pressure of the neighboring organs. The pyloric orifice is present in the midline or left when empty and markedly to the right when full [9]. The degree of the pylorus projected to the right side is worthy of attention, as it could be a sign of duodenal/pyloric strictures [3].

The current study showed the average length of the pyloric canal to be about 3 cm, in males 4.55±0.39 cm and females 3.56±.38 cm. According to Kirklin BR and Harris MT, it is 4.6 cm; they also reported that the increase in length might be due to the stretching of the lower part of the stomach, and the pyloric canal is elongated and narrowed in cases of pyloric stenosis [15]. West JB described the pyloric canal as a narrow passage of 3 cm in length, forming the lumen of the pyloric sphincter [16]. The pyloric canal and pylorus are synonymous and are a quarter of an inch, i.e., 6 mm, with a diameter varying from 0 to 6 mm, depending on the relaxation of the sphincter [17]. In the present study, the average diameter of the pyloric orifice was 7.7 mm. Similar findings were reported by the researcher [1]: 7.7±0.23 mm in males and 6.8±0.40 mm in females, with no significant difference.

In the present study, the average thickness of the pyloric orifice was 4.8 mm: 5.2±6.18 mm in males and 4.5±0.15 mm in females, with no significant difference. According to other authors like Crymble PT and Walmsleys T, it is 3.8-8.5 mm, as per Knight CD, it is 5.1 mm, and as per Keet AD and Heydenrych J, it is 7 mm [18,19,9]. Craver WL has noted in 13 fresh specimens that it was 4.4 mm, and in 10 fixed specimens, 7.1 mm [20].

In the present study, it was observed that the volume of the stomach is reduced (around 300 ml), while the previous studies mentioned it as 1000 ml [1]. The drawback of this study is that there is shrinkage of the stomach due to preservation in formalin, resulting in a reduction in capacity. Whang TS et al. reported that there is shrinkage of organs after preservation in formalin, which reduced the size significantly to 93% and 84%, respectively [21]. Arthur Frederick reported in his discussion that stomachs in live patients in the operation theatre and cadavers in the dissection hall do not differ much, as the atonicity/relaxation of the stomach after death and anesthesia are generally the same. He further reported that the differences between a formalin-filled stomach (used in the present study) and an active, live stomach could cause variations in results related to the structure of the pyloric end of the stomach; however, it is a generally accepted method, due to the absence of other means [4].

In the present study, 10% of the mucosa had either ulcers or perforations, and 90% were normal. In addition, 2% of the specimens had punched out ulcers 2x2 cm, circular in shape, along the LC, similar to the study of Robbins, who reported that 90% of the gastric ulcers are situated on the LC within the antrum or at the junction between the body and antral mucosa [22].

In the present study, 18% of the specimens showed less spaced rigidity at 2-3 mm, while 6% showed widely spaced rugosity. Gastric pits were found in 100% of the specimens. In 46% of the cases, the rugosity was straight in the region of the LC, similar to the findings of other researchers [7,23]. The thickening of the rugal folds is present in chronic hypertrophic gastritis and mesenteric disease. The normal width of the rugae is 2-4 mm, and a width of more than 5 mm is pathological [22]. Therefore, the absence of rugosity may be due to the underlying pathology, malignancy, or idiopathy.

Fetal morphological parameters of the stomach

In all our anthropometric measurements of the stomach in fetuses, GC, LC, and volume were influenced significantly by gestational age, which is similar to the study of Kepkep K et al. [24]. The length of the GC and LC varies according to gestational age [25,26]. The difference between the studies could be due to fewer fetuses involved in our study. Our study also lacks in correlating the size of the fetal stomach with the weight of the fetuses, as the fetuses were from pregnancies with a bad fetal outcome. The stomach's capacity is increased due to the growth of the left gastric wall [27]. The growth of omental bursa into the compound mesodermal analgen of the primitive right gastric wall and gastric mesenteries leads to the growth of the left gastric wall.

Nagata S et al. mentioned in their radiological study done on 618 fetuses that the capacity of the stomach had a linear increase with the growth of the stomach during 16-27 weeks of pregnancy; between 26 and 33 weeks, it was constant, in 33-36 weeks, it increased, and after 36 weeks it was reduced [27]. However, our study and the study done by Badiu CM et al. contradicted these findings, as we observed a linear growth of the stomach throughout pregnancy [28]. This difference could be due to the gastric peristaltic movements affecting the results.

## Conclusions

Stomach variations were found along the vertical and transverse axes, and shapes were classified as Type-I, Type II, and Type III, respectively. GC is significantly increased in males. A strong statistical correlation between GC, LC, and volume with the age of fetuses and the growth of the stomach is throughout the gestational age. This study is an attempt to add further data to the existing literature on how the dimensions of the stomach vary in adults and the growth of the stomach in fetuses according to their gestational age. This will help radiologists to identify congenital anomalies and help in the prediction of intrauterine growth retardation.
